# The Effect of Parity on the Quality of Colostrum of Holstein Dairy Cows in the Organic Production System

**DOI:** 10.3390/ani13030540

**Published:** 2023-02-03

**Authors:** Kinga Grodkowska, Marcin Gołębiewski, Jan Slósarz, Grzegorz Grodkowski, Piotr Kostusiak, Tomasz Sakowski, Marija Klopčič, Kamila Puppel

**Affiliations:** 1 Institute of Animal Science, Warsaw University of Life Sciences, Ciszewskiego 8, 02-786 Warsaw, Poland; 2 Institute of Genetics and Animal Biotechnology, Polish Academy of Science, Jastrzębiec, Postępu 36A, 05-552 Magdalenka, Poland; 3 Department of Animal Science, University of Ljubljana, SI-1230 Domžale, Slovenia

**Keywords:** colostrum, cow, immunoglobulin, density, age of cows, organic production

## Abstract

**Simple Summary:**

It should be emphasized that bovine placenta limits the transfer of immunoglobulins (Ig) between the mother and the fetus. Placental membranes are characterized by limited permeability, and only gases and small particles can penetrate through them; therefore, calves are born with minimal levels of antibodies. The quality of colostrum is shaped by environmental and genetic factors. It should be emphasized that the amount of colostrum produced is negatively correlated with its density and the basic chemical composition (protein, fat). So, its quality decreases as the amount of colostrum produced increases. Cows’ parity is also a modulating factor, and studies have shown an increase in colostral IgG corresponds with increasing parity. A special feature of organic production systems is the cows’ significantly lower productivity, which is adapted to environmental conditions. The research hypothesis proposes the verification of the statement: the organic system has a positive impact on the quality of colostrum. The aim of the study was to determine the effect of cows’ parity on the quality of colostrum, in an organic farm setting.

**Abstract:**

A special feature of organic production systems is the cows’ significantly lower productivity, which is adapted to environmental conditions. The quantity and quality of colostrum is negatively correlated, high amounts of colostrum are associated with low amounts of immunoglobulins. Cows’ parity is also a modulating factor, and studies have shown an increase in colostral IgG corresponds with increasing parity. This study’s aim was to determine the effect of cows’ parity on colostrum quality, in an organic farm setting. From a basic organic herd of dairy cattle, 40 Polish Holstein–Friesian cows were selected: 10 cows each of primiparous, second lactation, fourth lactation, and fifth lactation. Colostrum and transition milk samples were taken from each cow seven times: twice daily on the 1st and 2nd days after calving (every 12 h), and once daily on the 3rd to 5th days. Multiparous cows’ colostrum had higher levels of total proteins, casein, and non-fat dry matter, versus primiparous. Only cows in the second and fourth lactations produced very good quality colostrum (with immunoglobulins over 50 g/L), meeting standards for immunoglobulin concentration. In conclusion, the production of very good quality colostrum is limited during the first lactation, which may suggest the mammary gland is poorly developed, and, thus, immunoglobulin transport is limited. Variability in the colostrum’s immunoglobulin content from first and second collections post-calving was higher in multiparous versus primiparous cows. Therefore, it should be good practice to freeze colostrum from multiparous cows in case of poor-quality primiparous colostrum.

## 1. Introduction

Immunoglobulins (Ig) are extremely important elements in colostrum because they influence the immunization of the calf’s body [1,2,3]. Three classes of immunoglobulins have been identified in bovine colostrum: IgG (G1 and G2), IgM, and IgA. IgG makes up the largest proportion with 65−90%, whereas M class immunoglobulins make up 5%, and A class make up 15% [4]. In order for immunoglobulins to be fully used, attention is paid to such factors as the concentration and amount of colostrum administered, the time elapsed since birth (which is crucial due to a decreasing ability to absorb substances through the intestinal epithelium), and microbiological quality [4]. Colostrum that is defined as very good quality has an immunoglobulin content of 50 g/L [5,6]. However, Lombard et al. [7] proposed new standards which include four serum IgG categories: excellent, good, fair, and poor with serum IgG levels of ≥25.0, 18.0–24.9, 10.0–17.9, and <10 g/L, respectively.

Colostrum is synthesized in the mammary gland, mainly in the last 3 weeks before calving. During this period, IgG1 and IgG2 immunoglobulins are transported to the mammary gland, IgG1 passively, and IgG2 selectively [4]. The whole process of immunoglobulin transmission accelerates in the final stage of pregnancy when the udder also evolves within newly formed glandular epithelial cells [4]. The initiation of colostrum synthesis is closely related to a postpartum decrease in serum progesterone concentration and a short-term increase in estrogen concentration, as well as a slow but long-lasting release of prolactin into the blood [8].

Ahmann et al. [9] reported that the bovine placenta limits the transfer of immunoglobulins (Ig) between the mother and the fetus. Placental membranes are characterized by limited permeability, as only gases and small particles can penetrate through them; therefore, calves are born with minimal levels of antibodies [4]. One of the most difficult periods in the life of animals is their rearing, and the most vulnerable are the first days after birth. The most common causes of calf mortality are diarrhea (33–69%) and respiratory diseases (8–38%) [10]. The calves' ability to adapt to their environment is determined by their immune status [11]. Colostrum has antioxidant, antibacterial, and antiviral properties, and in addition, stimulates the immune and endocrine systems [12].

Organic production systems are not mentioned in the reports, which state that more than 60% of colostrum samples have reduced immunological quality [3]. A special feature of the organic production system is the cows’ significantly lower productivity, which is adapted to environmental conditions [4]. It should be emphasized that colostrum yield is negatively correlated with its density, i.e., its quality decreases as the amount of colostrum produced increases [13]. Cabral et al. [12] showed a negative correlation between the quantity and quality of colostrum, r = −0.42. The hypothesis of this research proposes the verification of the statement: the organic system has a positive impact on the quality of colostrum compared with conventional production cows, and that the age of the cows does not affect the quality of colostrum in an organic system. It should be noted that multiparous cows are exposed to antigens for a much longer period of time and therefore produce and pass higher amounts of antibodies to their colostrum.

The objective of this study was to determine the effect of cows’ parity on the quality of colostrum, in an organic farm setting, as well as to verify the statement that the colostrum from cows from organic farming systems is of very good quality.

## 2. Materials and Methods

### 2.1. Study Site and Study Animals

The cows were under veterinary control during the experiment. The animal study protocol was approved by the Second Ethics Committee for Animal Experimentation in Warsaw (protocol number WAWA2/086/2018). The experiment was carried out on a certified organic farm, on which a herd of approximately 300 cows was kept in a free-stall housing system, with the cows having an average productivity exceeding 6500 liters/cow/year. All cows selected for the experiment were healthy and showed no signs of *mastitis* or metabolic disorders. In addition, the somatic cell content of the last lactation before experiment was analyzed. After birth, the calves stayed with their mothers for 24 h, and then were moved to individual pens. From the basic herd, 40 Polish Holstein–Friesian cows were selected: 10 cows each of primiparous; second lactation; fourth lactation; and fifth lactation. Colostrum samples were collected over a 3-week period, in July 2022. Dry and fresh cows were fed according to the guidelines of the Nutrient Requirements Committee (Table 1). During the experiment, only 5 cows in the third lactation calved; therefore, the samples were not included in the analysis, as the group size was too small.

### 2.2. Data Collection

The colostrum and transition milk samples were taken individually from each cow 7 times during the experiment: twice a day on the 1st and 2nd days after calving (every 12 h), on the 3rd to 5th days once per day. The level of immunoglobulins is significantly reduced during the first 48 h; therefore, samples were taken twice a day during this period. Each colostrum container was coded and bore the following information: cow identification number (written down from the ear tag), date of collection (day, month, year), and sample number (1 to 7). Colostrum samples were taken using a bubble milking machine and then put into sterile plastic containers (250 mL) containing Milkstat CC preservative and were then stored at −20 °C until the planned analyses were carried out.

### 2.3. Chemical Analyses of Colostrum

The fat, protein, lactose, and casein as well as density were determined using a Milko-Scan FT-120 analyzer (Foss Electric, Hillerød, Denmark).

The determination of whey proteins was determined by RP-HPLC chromatography (Series 1100; Agilent Technologies Waldbronn, Germany) according to the methodology described by Puppel et al. [3]. Separations were performed at ambient temperature using solvent gradient with a C18 300A Jupiter column (Phenomenex, Torrance, CA, USA). The identification of peaks was confirmed by compared each peak’s retention time with that of injected reference standards (Sigma-Aldrich, St. Louis, MO, USA).

The determination of immunoglobulin G were determined by RP-HPLC chromatography (Series 1100; Agilent Technologies Waldbronn, Germany) according to the methodology described by Puppel et al. [3]. Separations were performed at ambient temperature using solvent gradient with a C18 300A Jupiter column (Phenomenex, Torrance, CA, USA). The identification of peaks was confirmed by compared each peak’s retention time with that of injected reference standards (Sigma-Aldrich, St. Louis, MO, USA).

The determination of fat-soluble vitamins and β-carotene were determined by RP-HPLC chromatography (Series 1100; Agilent Technologies, Waldbronn, Germany). Separations were performed at ambient temperature using solvent gradient: solvent A was methanol (Merck, Darmstadt, Germany) and water (Sigma-Aldrich, St Louis, MO, USA) in a ratio of 100:900 (*v/v*); and solvent B was water and methanol in a ratio of 900:100 (*v/v*), with a ZORBAX Eclipse XDB column (Agilent Technologies, Waldbronn, Germany). The total run time was 7 min, the flow rate was 1.2 mL/min, and the detection wavelength was 280 nm. The identification of peaks was confirmed by compared each peak’s retention time with that of injected reference standards (Sigma-Aldrich, St. Louis, MO, USA).

### 2.4. Statistical Analyses

The data were statistically analyzed using the multivariate least squares analysis of variance. The distribution of colostrum components was verified using the Shapiro–Wilk test. The tests were performed using IBM SPSS 22 software [14]. Only those interactions between factors whose impact was statistically significant (*p* ≤ 0.01 or *p* ≤ 0.05), as determined after preliminary statistical analyses, were included in the study. The following statistical model was used:

Y_ijk_ = μ + A_i_ + B_j_ + (A_i_ × B_j_) + e_ijk_

where: y is the dependent variable, µ is the overall mean, A_i_ is the fixed effect of the sample collection (I = 1 − 7), B_j_ is the fixed effect of the parity, A_i_ × B_j_ is the interaction between the subsequent sample collection effect and the parity, and e_ijk_ is the residual error.

## 3. Results and Discussion

It is well known that many factors influence colostrum quality. It has been proved that, in the main, the concentration of immunoglobulins in colostrum is determined by the following conditions: cow breed [15,16,17], calving season [18,19,20,21], nutrition during the dry period [22,23] and its duration, the incidence of mastitis [6], and lactation number [17,24,25,26].

**Figure 1:**
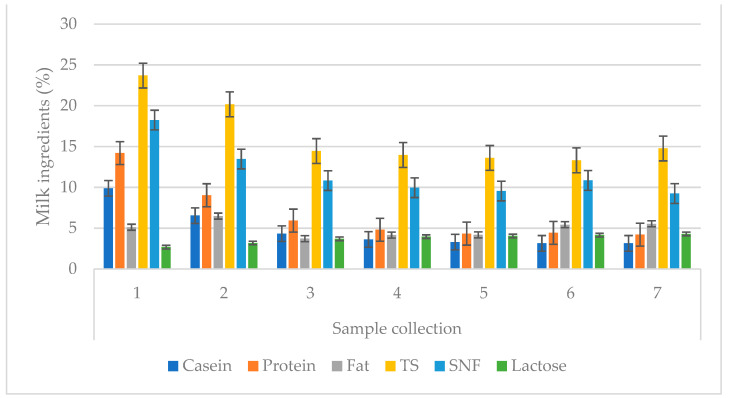
Changes in the chemical composition of colostrum over subsequent colostrum samples. Data are presented as least squares means along with their standard errors of mean. Statistical differences between collection groups at *p* ≤ 0.01. TS—total solid; SNF—solid non-fat.

Significant changes in composition may occur in cows over subsequent lactations. The research showed that cow colostrum from the first milking after calving was characterized by a relatively high protein content, after this, the protein percentage decreased. The stabilization of the protein levels was noticeable on the second day after birth (Figure 1). In the experiment, the average protein content of the first intake was 14.20%, which is a similar result to that obtained in a study conducted by Zachwieja [27], where the author gives the average protein content for primiparous cows as 14.65%, and for multiparous cows as 15.88%. However, the obtained result was higher than that given by Pawlak [28], where the protein was 13.97%. Soufleri et al. [26] also reported a significant (*p* < 0.05) increase in protein concentration in cows in their fourth lactation (18.96%) relative to cows in their first lactation (17.15%).

Casein content was at a level of 9.88% for the first milking, then it successively decreased to 3.14% in last collection; this result is higher than the one given by Lach [29], which was 5.08%. Additionally, Wąsowska and Puppel [30] proved that both the basic chemical composition and IgG composition in colostrum changes throughout the first six milkings postpartum.

Fat is a very important component in the diet of calves because it is a source of energy that is necessary to maintain body temperature [13,31]. The changes in fat content for subsequent intakes were not dependent; the proportion of fat was variable and did not follow a pattern, as was the case in studies by both Zachwieja [27] and Lach [29].

**Figure 2:**
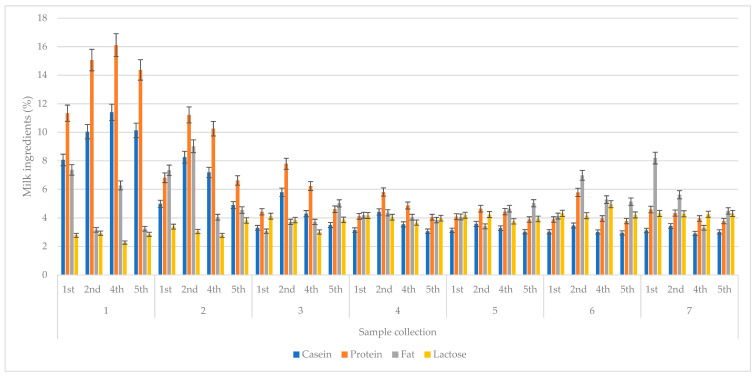
Changes in the basic chemical composition of colostrum (%) for subsequent collections, depending on parity. Data are presented as least squares means along with their standard errors of mean. Statistical differences between collection groups at *p* ≤ 0.01.

The dry matter content consistently decreased until the sixth collection, but in the seventh it was noticeably increasing, which was due to the increased fat and lactose. Almost all of the above-mentioned components were characterized by a tendency for their content level in the colostrum to decrease over the course of the collections; the exception was lactose, whose content increased with time after birth. (Figure 1). This does not differ significantly from the levels this parameter given by Zachwieja [27], where it was 2.56% and 3.89%, respectively. The highest protein content was characteristic of the cows’ colostrum in the fourth lactation, i.e., 16.11% (Figure 2). The highest fat content was found to be in the second collection of colostrum during the second lactation (Figure 2). The values and correlations that characterized these two parameters are in line with Zachwieja [27], who stated, moreover, that the fat content for each subsequent lactation during the first samples just after calving was at increasingly lower levels, which was not confirmed by the current experiment. On the other hand, the casein content was similar to that of protein for the first collection, where, in cows, it was at its highest level in the fourth lactation. However, the share of casein in subsequent collections was highest for the second lactation. The lactose content in the first collection was lowest in cows in the fourth lactation (Figure 2). Quinn et al. [32] observed changes in the oligosaccharide profile during the first days of lactation. They found an increase in oligosaccharide and lactose concentrations during the days following postpartum. The lowest concentrations were observed in the colostrum, whereas the highest were in the mature milk. Changes in the concentration of oligosaccharides in colostrum according to lactation number were also reported: the higher the lactation number, the higher the concentration of oligosaccharides.

On the other hand, Soufleri et al. [26] reported that the lowest concentration of lactose was found in colostrum from multiparous cows in the fourth lactation. Zarei et al. [33] reported significantly higher fat levels during the first parity than in others. Moreover, the highest protein concentration was observed in cows past their third parity. Furthermore, Kessler et al. [34] reported higher fat and lactose concentration in primiparous cows (*p* < 0.0001) than multiparous cows. Aydogdu and Guzelbektes [35] obtained the same results. The average fat concentration in the colostrum of Holstein–Friesian cows decreased with an increasing lactation number. The colostrum from primiparous cows contained an average of 7.46% fat, whereas that of multiparous cows was only 5.44%. Jolazadeh et al. [36] reported the effects of parity on the fatty acid profile of colostrum. Their study showed that colostrum from primiparous cows had higher concentrations of C18:0 and lower concentration of C16:0 compared to multiparous cows. This may be related to differences in energy requirements and partitioning, as well as differences in FA synthesis between primiparous and multiparous cows. Analogous results were obtained by Garcia et al. [37] and O'Callaghan et al. [38].

**Figure 3:**
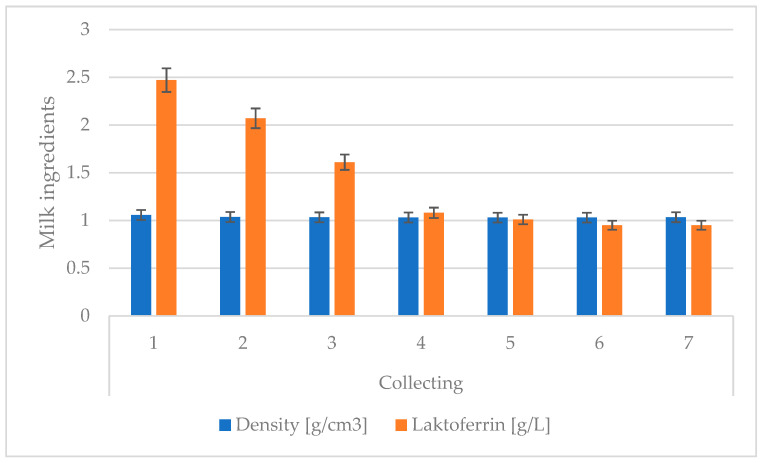
Changes in lactoferrin and density values for colostrum over subsequent colostrum samples. Data are presented as least squares means along with their standard errors of mean. Statistical differences between collection groups at *p* ≤ 0.01.

The colostrum’s density was on average 1.057 g/cm^3^ for the first collection, then consistently decreased to 1.029 g/cm^3^ for the fifth, sixth, and seventh collections. A slight increase was observed in relation to the previous four collections (Figure 3 and Table 2). The colostrum’s density can be described as satisfactory for the tested cows, because, according to Demkowicz [39], it should be 1.058 g/cm^3^, so it does not differ significantly from the data in the literature. The average lactoferrin (LF) content in the cows’ colostrum for the first collection was 2.47 g/L and 0.95 g/L for the 5th day of the experiment. The demonstrated lactoferrin concentration was higher than the literature’s data, where colostrum content was given as 1 g/L. In the present study, an increase was observed in the average concentration of LF in colostrum from cows that were in their second lactation relative to colostrum from cows that were primiparous. The average concentrations of LF in colostrum from cows in their fourth and fifth lactations were higher than the average concentrations of LF for primiparous cows but did not exceed the concentration from cows in their second lactation. Bar et al. [24] reported increase concentration of LF with increased parity from 28.62 mg/100 mL in the first lactation to 45.25 mg/100 mL in the fourth lactation.

**Figure 4:**
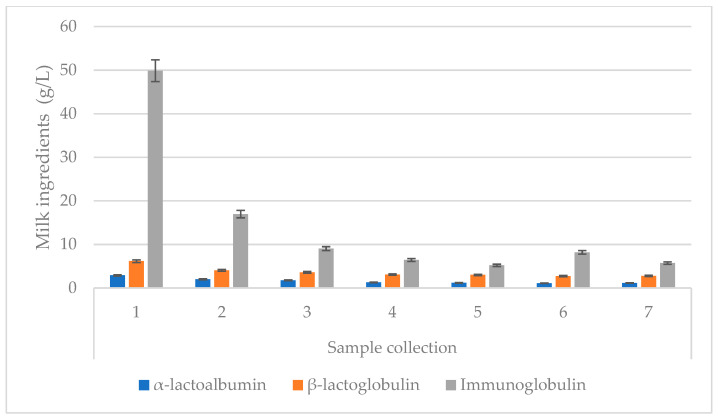
Changes in the whey protein values of colostrum for subsequent colostrum samples. Data are presented as least squares means along with their standard errors of mean. Statistical differences between collection groups at *p* ≤ 0.01.

The experiment showed that the α-lactalbumin content in the cows’ colostrum for the first collection was 2.90 g/L, and that it decreased successively until the sixth collection, followed by a minimal increase in concentration in the seventh collection. The average content of β-lactoglobulin in the conducted experiment for the first collection was 6.16 g/L, whereas the seventh produced a content of 2.78 g/L. The study also showed that the period of time that had passed since birth had a significant influence on the development of immunoglobulin levels. The average immunoglobulin content in the colostrum derived from the first collection was close to 50 g/L, but by the second collection it had already fallen by over 60%. On day 5 of the experiment the Ig content was 5.71 g/L (Figure 4).

The research shows that in the first collection, the colostrum sample with the richest α-lactalbumin levels were for cows in their fourth lactation. On the other hand, the colostrum for the fifth lactation in the first collection was characterized by the highest β-lactoglobulin content. Szulc and Zachwieja [7] claimed that the younger the cows, the lower the colostrum’s immunoglobulin content, which does not fully coincide with the current experiment. In the first collection, cows in both the second lactation and fifth lactations showed high concentrations of immunoglobulins (Table 3). The greatest decrease in the proportion of immunoglobulins in the colostrum during the 1st day after calving was in cows in their fifth lactation. For the second and fourth lactations, the decrease was greater than for cows in their first lactation. The obtained results confirm the observations of Zachwieja [27], which indicated higher levels of immunoglobulins in multiparous cows in comparison to the primiparous cows and coincide with the author's thesis that a slower decrease in Ig is characteristic for older cows (compared cows with the highest Ig content in the first collection). Another statement in Zachwieja’s [27] research was proved in a study by Guliński et al. [40] which stated that cows in their second and subsequent lactations showed a higher percentage of immunoglobulin. The average immunoglobulins yield for fresh colostrum covers a wide range of results: from 26.50 g/L to 79.43 g/L (Table 2). Additionally, Puppel et al. [3] reported that colostrum with immunoglobulins over 50 g/L had significantly higher concentrations of lactoferrin and immunoglobulin G.

A strong correlation between immunoglobulin concentration and lactation number was also reported by Kessler et al. [34]. Colostrum from multiparous cows had significantly (*p* < 0.0001) higher IgG concentration relative to primiparous cows. The same correlation was demonstrated by Aydogdu and Guzelbektes [35]. Zarei et al. [33] reached different conclusions, finding no significant increase in IgG and IgM concentration with increasing parity; the authors only pointed out the presence of an increasing trend in relation to the immunoglobulin concentration relative to an increase in parity. IgG concentration ranged from 35.6 mg/mL in the second lactation to 37.53 mg/mL in the fourth and subsequent lactations.

**Figure 5:**
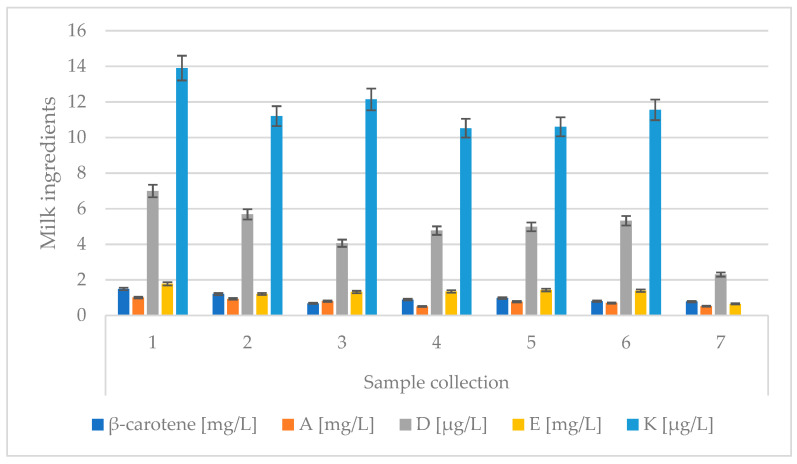
Changes in vitamin values in colostrum for subsequent colostrum samples. Data are presented as least squares means along with their standard errors of mean. Statistical differences between collection groups at *p* ≤ 0.01.

The total vitamin content in the colostrum was highest for the first collection. The level of β-carotene was 1.49 mg/L; vitamin A, 1.01 mg/L; vitamin D, 6.99 µg/L; vitamin E, 1.78 mg/L; and vitamin K, 13.90 µg/L (Figure 5). The vitamin E content was almost 10 times lower compared to data provided by Szulc and Zachwieja [7]; according to the authors, the level of this vitamin in the colostrum was 10 g. It can be concluded that the cows’ dietary ration, which included in the experiment, was poor in vitamin E. The transfer of vitamin A and vitamin E across the placenta is limited. To avoid the deficiencies and diseases occurring, it is important that calves receive colostrum that is high in these vitamins [41].

Due to dried cows' insufficient nutrition in terms of vitamin E content, it is necessary to enrich the first intake of colostrum with 1 g of this vitamin [42]. According to Kowalski [42], this is very important for calves because vitamin E is responsible for the state and activity of neutrophilia. The vitamin E content in colostrum depends, to a large extent, on the cows' diet, but there is also considerable variation between cows in this respect. The vitamin A content also deviated from both the standards given in the literature (1.8 g/L), and the results in the conducted experiment (1.01 g/L). The vitamin A content in the ration is reflected by its content in the cows’ blood. Vitamin A, unlike vitamin E, easily crosses the placental barrier, so that the calf accumulates it in its liver. Moreso, according to Kowalski [42], its level in colostrum is not so important that the calf covers its needs.

The highest content of β-carotene was found in the first and second collections and was characterized by cows in their fifth lactation. According to research, the colostrum with the highest abundance of vitamin A and E, for cows immediately after calving, was from cows in their first and second lactation. The lactating cows had colostrum with the richest levels of vitamin D, whereas the colostrum from the youngest cows was the most abundant in vitamin K (Table 4). Vitamin E from each of the seven collections was at a similar level for both the fourth and fifth lactations. The greatest dynamics in β-carotene change for the second collection were observed in cows in their second lactation (0.5 mg), vitamin A—elements (0.2 mg), vitamin D—also elements (3.11 µg), vitamin E—cows in the second lactation (1.22 mg), and vitamin K (4.64 µg).

According to the literature, the average concentration of vitamin A in colostrum is 2.33–3.69 mg/L [41,43,44].

## 4. Conclusions

On the basis of this experiment, it was found that there were significant differences in the levels of the colostrum’s bioactive components with immunostimulating properties, which was due to how long after calving colostrum was ingested and the significant differences in the level of these components due to the parity of cows. Studies have shown that there is also a colostrum quality problem in an organic production system, where more than 40% of colostrum samples have reduced immunological quality, with immunoglobulins below 50 g/L in the first collection. Only cows in their second and fourth lactation showed very good colostrum quality, meeting the standards in terms of concentration of immunoglobulins. Multiparous cows had colostrum that was distinguished by a higher level of total protein, casein, and non-fat dry matter, compared to primiparous cows. On the other hand, colostrum from primiparous cows was characterized by a higher content of colostrum dry matter in relation to multiparous cows. Variability in the immunoglobulin content of the colostrum obtained in the first and second collections, after calving, was higher in multiparous cows than in primiparous cows. Ig concentrations in colostrum is correlated with the number of lactations. Older cows produce colostrum with higher Ig levels; this may be because older cows have been exposed to antigens for a longer time than younger cows. Therefore, it should be considered good practice to freeze colostrum from multiparous cows in case of poor-quality primiparous colostrum. It can be concluded that the production of good quality colostrum is limited in the first lactation, which may suggest poor development of the mammary gland, and thus limited transport of immunoglobulins.

## Figures and Tables

**Table 1:** Ingredient and chemical composition of cows diet.

Trait	Cows	
	Close-Up	Fresh
Feed intake and energy balance		
Conserved forages (kg DM/d)	11.5	12.5
Concentrate (kg DM/d)	1.9	4.5
Total (kg DM/d)	13.4	17.0
NEL intake (MJ NEL/d)	74.3	84.9
EB (MJ NEL/d)	18.7	30.1
nXP intake (g/d)	2232	2658
BW (kg)	675	659

nXP = utilizable crude protein at the duodenum.

**Table 2:** Changes in lactoferrin and density values for colostrum over subsequent collection, depending on parity. Data are presented as least squares means. Means marked with the same letters differ significantly at: lowercase letters, *p* ≤ 0.05; uppercase letters, *p* ≤ 0.01.

Sample Collection	Lactation	Ingredient	
		Density [g/cm^3^]	Lactoferrin [g/L]
1	1	1.037 ^ABC^	1.69 ^ABC^
	2	1.056 ^ADE^	3.32 ^ADE^
	4	1.049 ^BDf^	2.05 ^BDF^
	5	1.051 ^CEf^	3.20 ^CEF^
2	1	1.029 ^ABC^	0.92 ^ABC^
	2	1.039 ^ADE^	3.03 ^ADE^
	4	1.040 ^BDF^	2.68 ^BDF^
	5	1.036 ^CEF^	1.25 ^CEF^
3	1	1.031 ^A^	0.66 ^ABC^
	2	1.042 ^ABC^	2.40 ^ADE^
	4	1.031 ^B^	2.06 ^BDF^
	5	1.031 ^C^	1.03 ^CEF^
4	1	1.032 ^a^	0.62 ^ABC^
	2	1.036 ^abc^	2.10 ^ADE^
	4	1.032 ^b^	0.69 ^BDF^
	5	1.031 ^c^	0.79 ^CEF^
5	1	1.031 ^a^	0.65 ^AB^
	2	1.035 ^abc^	1.90 ^ACD^
	4	1.030 ^b^	0.63 ^CE^
	5	1.029 ^c^	0.72 ^BDE^
6	1	1.031 ^ab^	0.58 ^AB^
	2	1.028 ^ac^	1.83 ^ACD^
	4	1.029 ^b^	0.58 ^CE^
	5	1.030 ^C^	0.65 ^BDE^
7	1	1.023 ^ABC^	0.56 ^AbC^
	2	1.031 ^A^	1.90 ^ADE^
	4	1.032 ^B^	0.52 ^BDF^
	5	1.031 ^C^	0.73 ^CEF^

**Table 3:** Changes in the whey protein values of colostrum for subsequent collections, depending on parity. Data are presented as least squares means. Means marked with the same letters differ significantly at: lowercase letters, *p* ≤ 0.05; uppercase letters, *p* ≤ 0.01.

Sample Collection	Lactation	Whey Protein		
		α-Lactalbumin [g/L]	β-Lactoglobulin [g/L]	Immunoglobulin G [g/L]
1	1	1.24 ^ABC^	5.27 ^ABC^	26.50 ^ABC^
	2	3.29 ^Ade^	6.19 ^AD^	79.43 ^ADE^
	4	3.71 ^Bd^	6.20 ^BE^	38.14 ^BDF^
	5	3.52 ^Ce^	8.83 ^CDE^	69.32 ^CEF^
2	1	0.94 ^ABC^	2.37 ^ABC^	14.37 ^ABC^
	2	2.85 ^ADE^	4.94 ^A^	19.51 ^ADE^
	4	1.79 ^BDF^	4.92 ^B^	22.55 ^BDF^
	5	3.12 ^CEF^	4.58 ^C^	8.64 ^CEF^
3	1	0.75 ^ABC^	3.08 ^ABC^	6.86 ^ABC^
	2	2.30 ^Ade^	3.97 ^A^	11.70 ^AD^
	4	1.94 ^BdF^	3.92 ^B^	11.83 ^BE^
	5	2.57 ^CeF^	3.78 ^C^	3.95 ^CDE^
4	1	0.69 ^ABC^	2.80 ^AB^	5.65 ^ABC^
	2	2.14 ^ACd^	3.63 ^AC^	6.30 ^ADE^
	4	0.77 ^CE^	3.19 ^BD^	8.84 ^BDF^
	5	1.92 ^BdE^	2.83 ^CD^	4.50 ^CEF^
5	1	0.75 ^AB^	3.17 ^A^	5.14 ^ABC^
	2	1.92 ^ACD^	3.24 ^B^	3.81 ^ADE^
	4	0.72 ^CE^	3.01 ^C^	7.46 ^BDF^
	5	1.79 ^BDE^	2.63 ^ABC^	4.47 ^CEF^
6	1	0.66 ^AB^	2.82 ^abC^	4.68 ^ABC^
	2	1.77 ^ACD^	3.05 ^adE^	16.89 ^ADE^
	4	0.72 ^CE^	2.73 ^bd^	5.79 ^BDF^
	5	1.63 ^BDE^	2.40 ^CE^	3.81 ^CEF^
7	1	0.63 ^AB^	2.72 ^Abc^	11.12 ^ABC^
	2	2.00 ^ACd^	3.37 ^ADE^	1.97 ^ADE^
	4	0.59 ^CE^	2.56 ^bDf^	5.63 ^BDF^
	5	1.82 ^BdE^	2.67 ^cEf^	3.57 ^CEF^

**Table 4:** Changes in vitamin values in colostrum for subsequent collections, depending on parity. Data are presented as least squares means. Means marked with the same letters differ significantly at: lowercase letters, *p* ≤ 0.05; uppercase letters, *p* ≤ 0.01.

Sample Collection	Lactation	Vitamin				
		β-Carotene [mg/L]	A [mg/L]	D [µg/L]	E [mg/L]	K [µg/L]
1	1	1.19 ^ABC^	0.74 ^ABC^	5.90 ^ABC^	1.62 ^Abc^	14.78 ^AB^
	2	1.71 ^ADE^	1.33 ^ADF^	6.52 ^ADE^	2.77 ^ADE^	14.18 ^CD^
	4	1.34 ^BDF^	1.00 ^BDg^	8.04 ^BDF^	1.14 ^bD^	12.87 ^ACF^
	5	1.99 ^CEF^	0.94 ^CFg^	7.99 ^CEF^	1.17 ^cE^	13.56 ^BDF^
2	1	0.97 ^abC^	0.54 ^ABC^	5.48 ^ABC^	1.07 ^a^	11.04 ^ABC^
	2	1.19 ^aD^	1.34 ^ADF^	3.41 ^ADE^	1.55 ^abc^	9.51 ^AD^
	4	1.16 ^bE^	0.90 ^BD^	5.68 ^BDF^	1.05 ^b^	13.67 ^BDE^
	5	1.80 ^CDE^	0.93 ^CF^	10.37 ^CEF^	1.15 ^c^	9.97 ^CE^
3	1	0.61 ^ABC^	0.47 ^ABC^	3.02 ^ABC^	1.08 ^A^	7.21 ^ABC^
	2	1.43 ^ADE^	1.34 ^ADE^	8.02 ^ADE^	2.30 ^ABC^	11.98 ^ADE^
	4	0.13 ^BDF^	0.58 ^BDF^	1.30 ^BDF^	0.83 ^B^	16.75 ^BDF^
	5	0.40 ^CEF^	0.90 ^CEF^	3.75 ^CEF^	0.80 ^C^	13.09 ^CEF^
4	1	0.60 ^BCb^	0.49 ^ABC^	4.45 ^ABC^	0.68 ^A^	5.98 ^ABC^
	2	0.72 ^aBeF^	0.66 ^ADE^	1.74 ^ADE^	0.62 ^BC^	15.66 ^ADE^
	4	0.82 ^CeG^	0.39 ^BDF^	8.89 ^BDF^	1.29 ^ABD^	6.78 ^BDF^
	5	1.67 ^DFG^	0.50 ^CEF^	3.30 ^CEF^	2.29 ^CD^	16.83 ^CEF^
5	1	0.43 ^ABC^	0.58 ^ABC^	0.99 ^ABC^	0.73 ^ABc^	11.84 ^AB^
	2	0.90 ^ADE^	0.82 ^ADE^	2.31 ^ADE^	1.04 ^ADE^	11.97 ^CD^
	4	1.51 ^BDF^	0.97 ^BDF^	12.58 ^BDF^	2.77 ^BDF^	9.88 ^ACE^
	5	1.20 ^CEF^	0.70 ^CEF^	3.09 ^CEF^	0.94 ^cEF^	6.87 ^BDE^
6	1	0.35 ^ABC^	0.47 ^AbC^	1.25 ^ABC^	0.71 ^ABc^	9.18 ^ABC^
	2	1.27 ^ADE^	0.87 ^ADE^	9.14 ^ADE^	2.10 ^ADE^	14.71 ^ADE^
	4	0.99 ^BDF^	0.51 ^bDF^	6.47 ^BDF^	1.68 ^BDF^	10.95 ^BDF^
	5	0.42 ^CEF^	1.15 ^CEF^	3.50 ^CEF^	0.77 ^cEF^	11.19 ^CEF^
7	1	0.58 ^AB^	0.42 ^ABC^	2.04 ^ABC^	0.94 ^aBC^	5.84 ^ABC^
	2	0.88 ^ACD^	0.74 ^ADE^	1.98 ^ADE^	0.68 ^ade^	11.16 ^ADE^
	4	0.57 ^CE^	0.39 ^BDF^	1.74 ^BD^	0.48 ^Bd^	10.13 ^BDF^
	5	1.16 ^BDE^	0.53 ^CEF^	4.68 ^CE^	0.40 ^Ce^	6.86 ^CEF^

## Data Availability

All data generated or analyzed during the study are included in this published article. The datasets used and/or analyzed in the current study are available from the corresponding author on reasonable request.
